# *Cox4i2*, *Ifit2*, and *Prdm11* Mutant Mice: Effective Selection of Genes Predisposing to an Altered Airway Inflammatory Response from a Large Compendium of Mutant Mouse Lines

**DOI:** 10.1371/journal.pone.0134503

**Published:** 2015-08-11

**Authors:** Marion Horsch, Juan Antonio Aguilar-Pimentel, Clemens Bönisch, Christophe Côme, Cathrine Kolster-Fog, Klaus T. Jensen, Anders H. Lund, Icksoo Lee, Lawrence I. Grossman, Christopher Sinkler, Maik Hüttemann, Erwin Bohn, Helmut Fuchs, Markus Ollert, Valérie Gailus-Durner, Martin Hrabĕ de Angelis, Johannes Beckers

**Affiliations:** 1 Helmholtz Zentrum München GmbH, German Mouse Clinic, Institute of Experimental Genetics, D-85764 Neuherberg, Germany; 2 Department of Dermatology and Allergy, TUM and Clinical Research Division of Molecular and Clinical Allergotoxicology, Munich, Germany; 3 Biotech Research and Innovation Centre, Lund Group, University of Copenhagen, Ole Maaloes vej 5, DK-2200 Copenhagen, Denmark; 4 College of Medicine, Dankook University, Cheonan-si, Chungcheongnam-do, 330–714, Republic of Korea; 5 Wayne State University, Center for Molecular Medicine and Genetics, 540 E. Canfield Avenue, Detroit, Michigan 48201, United States of America; 6 Universitätsklinikum Tübingen, Institut für Medizinische Mikrobiologie, Elfriede-Aulhorn-Strasse 6, D-72076 Tübingen, Germany; 7 Center of Allergy and Environment Munich (ZAUM), Technische Universität München, Munich, Germany; 8 Department of Infection and Immunity, Luxembourg Institute of Health, Esch-sur-Alzette, Luxembourg and Department of Dermatology and Allergy Center, Odense Research Center for Anaphylaxis, University of Southern Denmark, Odense, Denmark; 9 Technische Universität München, Chair of Experimental Genetics, D-85354 Freising, Germany; 10 German Center for Diabetes Research (DZD), D-85764 Neuherberg, Germany; French National Centre for Scientific Research, FRANCE

## Abstract

We established a selection strategy to identify new models for an altered airway inflammatory response from a large compendium of mutant mouse lines that were systemically phenotyped in the German Mouse Clinic (GMC). As selection criteria we included published gene functional data, as well as immunological and transcriptome data from GMC phenotyping screens under standard conditions. Applying these criteria we identified a few from several hundred mutant mouse lines and further characterized the *Cox4i2^tm1Hutt^*, *Ifit2^tm1.1Ebsb^*, and *Prdm11^tm1.1ahl^* lines following ovalbumin (OVA) sensitization and repeated OVA airway challenge. Challenged *Prdm11^tm1.1ahl ^*mice exhibited changes in B cell counts, CD4^+^ T cell counts, and in the number of neutrophils in bronchoalveolar lavages, whereas challenged *Ifit2^tm1.1Ebsb^* mice displayed alterations in plasma IgE, IgG1, IgG3, and IgM levels compared to the challenged wild type littermates. In contrast, challenged *Cox4i2^tm1Hutt^* mutant mice did not show alterations in the humoral or cellular immune response compared to challenged wild type mice. Transcriptome analyses from lungs of the challenged mutant mouse lines showed extensive changes in gene expression in *Prdm11^tm1.1ahl^* mice. Functional annotations of regulated genes of all three mutant mouse lines were primarily related to inflammation and airway smooth muscle (ASM) remodeling. We were thus able to define an effective selection strategy to identify new candidate genes for the predisposition to an altered airway inflammatory response under OVA challenge conditions. Similar selection strategies may be used for the analysis of additional genotype – envirotype interactions for other diseases.

## Introduction

In combination with environmental factors, genetic predisposition may either promote disease susceptibility or protect from it [1]. Several studies analyzed the immune response in distinct mouse models under steady-state and environmental challenge conditions [2, 3] and profiled gene expression in rodents exposed to different environmental agents [4, 5] including ovalbumin (OVA) [6–9]. Transcriptional changes monitored in mouse lungs following OVA challenge were associated with immune response, including chemokines and proteases, TGFβ signaling, and metabolic and oxidative stress responses [6, 9–11]. Further animal models are needed to better understand the pathological processes of the airway inflammatory response. The identification of additional genes involved in the immune response to allergen exposure will further our understanding of the role of specific gene-environment interactions and lay the basis for new diagnostic and therapeutic approaches.

Systemic primary phenotyping in the German Mouse Clinic (GMC) is designed to identify affected organs in mutant mouse lines utilizing standardized and broad-based phenotypic screening methods [12–14]. The primary phenotyping panel also provides original data from allergy and immunology screens under standard husbandry conditions [15]. This enables browsing of large phenotype data sets of hundreds of mutant mouse lines to identify new potential inflammatory models. However, the genetic predisposition for diseases is often only revealed if environmental factors challenge the organism [16, 17]. Such environmental challenge assays demand specific and reproducible experimental interventions and more sophisticated phenotyping methods that are in most cases impossible to apply in a high-throughput, primary phenotyping setting. Thus we established an effective selection strategy for the identification of new genetic models that are predisposed to an altered airway inflammatory response. The selection of such mouse models is based on pre-defined criteria that include both published gene functional annotation and GMC primary phenotyping data. In addition, we chose transcriptomics of lungs as a highly sensitive tool to identify subtle changes at the gene expression level [18]. Transcriptomics is also instrumental to study gene regulatory mechanisms during the induced airway inflammatory process.

For the proof-of-concept we selected three out of more than 300 mutant mouse lines that were previously phenotyped in the primary GMC screens. We specifically selected mutant mouse lines that did not show any changes of immunological parameters under standard conditions. The selected mutant mouse lines carry loss-of-function alleles in either *cytochrome c oxidase subunit 4 isoform 2* (*Cox4i2*) [19], *interferon-induced protein with tetratricopeptide repeats 2* (*Ifit2*) [20], or *PR domain containing 11* (*Prdm11*) [21, 22]. To induce an airway inflammatory response, the mutant mouse lines were sensitized via intraperitoneal injections of ovalbumin (OVA) followed by inhalative exposures to OVA aerosol [23], which has been shown to faithfully model the allergic response in humans [24, 25]. The inflammatory and immunological response in the three OVA challenged mutant mouse lines was analyzed in lungs, bronchoalveolar lavages (BAL), and in blood plasma. For the analysis of transcriptomes, we focused on gene expression changes in lung as the primary target organ of the inhaled allergen in the OVA challenged mice [26]. Moreover, the lung is the site of cellular defense via alveolar macrophages and it triggers the humoral response via secretion of immunoglobulins and chemokines.

Our phenotyping results under OVA airway challenge conditions revealed genotype-specific alterations of plasma IgE and other immunoglobulin isotype levels (*Ifit2* mutant line), B and T cell quantity (*Prdm11* mutant line), and gene expression patterns in lung (*Cox4i2*, *Ifit2*, and *Prdm11* mutant lines). In particular, gene expression changes following OVA challenge in all three mutant mouse lines suggested altered airway smooth muscle (ASM) remodelling in comparison to challenged wild type littermates.

## Materials and Methods

### Ethics statement and mouse lines

All mice were housed under specific pathogen free conditions in accordance to FELASA guidelines. Mouse husbandry and all animal experiments were carried in accordance with German legal guidelines and following the approval (approval number 55.2-1-54-2532-144-10) of the responsible animal welfare authorities and the ethics board of the district government of Upper Bavaria, Germany (full name: Regierung von Oberbayern, Sachgebiet 54). In this study we analyzed the homozygous knockout models *Cox4i2* ^*tm1Hutt*^ [19], *Ifit2* ^*tm1*.*1Ebsb*^ [20], and *Prdm11* ^*tm1*.*1ahl*^ [21, 22]. The *Cox4i2* ^*tm1Hutt*^ strain was back-crossed to the C57BL/6 background for 12 generations. The *Ifit2* ^*tm1*.*1Ebsb*^ and *Prdm11* ^*tm1*.*1ahl*^ alleles were generated in ES cells of the 129/Ola background and subsequently backcrossed to the C57BL/6 strain for at least 5 and 7 generations, respectively. For each mutant mouse line an age-matched cohort of wild type littermates on the respective genetic background was used as reference group. S1 Table summarizes information regarding mutant alleles, gender, age, and group sizes.

### OVA airway challenge

For allergen sensitization and challenge, mice were treated twice by intraperitoneal injection of 10 μg OVA (Ovalbumin, Sigma, Germany) and 2 mg alum (inject-Alum Pierce, Rockford, USA) dissolved in 200 μl phosphate buffered saline (PBS) at day (D) 1 and D7 as previously described [25, 27]. Four weeks after sensitization mice were challenged three times by inhalative exposure to OVA aerosol (1% in PBS) for 30 min once a day (D30-D33-D36). 24 h after the last challenge (D37) blood samples were collected and animals sacrificed to obtain bronchoalveolar lavage (BAL) samples. In order to perform transcriptome analyses, lungs of challenged and unchallenged mice of each mouse line were collected (S1 Table).

### Quantification of immunoglobulin in blood plasma and cytokines in BAL

Mouse blood samples were collected before and after challenge as previously described [25, 28, 29] by puncturing the retro-orbital plexus (Li-heparin-coated tubes, KADE, Nümbrecht, Germany) under isoflurane anesthesia. Blood samples were centrifuged (10 min, 5000 x g) in a refrigerated centrifuge to separate cells and plasma. Plasma total IgE was measured using a classical immunoassay isotope-specific sandwich ELISA. In brief, plasma samples and standards for murine IgE (Mouse IgE, K clone C38-2; BD Pharmingen, Heidelberg, Germany) were transferred to microtiter plates coated with 10 μg/ml anti-mouse-IgE rat monoclonal IgG (clone-PC 284; The Binding Site, Schwetzingen, Germany). As secondary antibody, 2.5 μg/ml of biotinylated monoclonal rat anti-mouse IgE (clone R35-118; BD Pharmingen) was used, followed by incubation with DB OptEIA Reagent Set B (BD Pharmingen). Signal intensities of labeled total murine IgE were measured at 450 nm in a standard microwell ELISA reader and results reported in ng/ml based on a standard curve of purified IgE. The determination of other immunoglobulin isotype levels was performed with a combined multiplexed bead-based assay system (Biorad, USA). Briefly, diluted blood plasma was stained with a bead mixture containing Luminex beads of five different regions coupled with antibodies specific for mouse IgG1, IgG2a, IgG3, IgM, and IgA (BD Pharmingen; Bio Plex Bead coupling Kit; Biorad, CA, USA). After streptavidin-PE was added to the plates, samples were measured and results obtained on a Bio-Plex reader [25]. For cytokine measurement in BAL, 50μl of undiluted sample was stained using the mouse Cytokine Th1/Th2 Bio-Plex Pro panel multiplex kit (Bio-Rad Laboratories, Munich, Germany).

### Quantification of cell populations in BAL

Flow cytometry was performed to quantify cell surface marker staining of BAL cells using the following monoclonal antibodies: anti-CD8a, anti-Ly6c, anti-CD4, anti-CD62l, anti-CD3, anti-CD25, anti-CD193, anti-NK1, anti-Gr1, anti-CD19, anti-CD11b, anti-CD11c, and anti-MHC-II [25]. Data were acquired using a LSRII flow cytometer (BD Bioscience) and further analyzed with FACSDiVa (BD Bioscience) and Flowjo V.7.2.2 (Tree star, Ashland, USA) software.

### T and B cell stimulation

Splenocytes of *Ifit2* ^*tm1*.*1Ebsb*^ and *Prdm11* ^*tm1*.*1ahl*^ mutant and wild type littermates (males, n = 5, 7–12 weeks of age) were isolated and enriched for naȉve B cells by MACS using the B cell isolation kit (Miltenyi). Purity of B cells was controlled by FACS analysis (B220 staining, and CD4 and CD8 T cell control staining). Both total cells and purified B cells were cultivated in 200 μl/well RPMI complete plates (100,000 cells/well). For T cell stimulation total cells were incubated 4 days with 20 U/ml IL-2 and anti-CD3 in varying concentrations (0.05, 0.5 and 5.0 μg/ml). Purified B cells were stimulated 8 days by incubation with 1 μg/ml anti-CD40 and 1 ng/ml IL-4 or 5 μg/ml anti-CD40 and 10 ng/ml IL-4. The number of viable cells was determined by CellTiter-Glo Assay (Promega) and IgE concentration in the supernatant was measured by ELISA.

### Statistics

If not otherwise indicated significant differences were calculated using the Mann-Whitney rank-sum test.

### Transcriptome profiling of lung

Lungs of mutant and wild type female mice were dissected from all cohorts (S1 Table) and total RNA was extracted according to a standardized protocol (RNeasy mini kit, Qiagen). Using the Illumina TotalPrep RNA Amplification kit (Ambion) 500 ng of high quality total RNA was amplified and hybridized to MouseRef-8v2 Expression BeadChips (Illumina, Sand Diego, CA, USA). Hybridization, staining, and scanning were done according to the manufacturer’s protocol. Data were subjected to cubic spline normalization and background subtraction (Genomestudio V2011.1, Illumina) and SAM (Significant Analysis of Microarray [30, 31]) was used to identify differentially expressed genes, as recently described [18] (false discovery rate (FDR) < 10%, fold change > 1.7). Hierarchical cluster analysis [32] was used to identify similar gene expression patterns applying the average-linkage-method as distance and the Euclidean distance as distance-metric.

Over-represented functional annotations among differentially expressed genes were provided as Gene Ontology (GO) terms (Ingenuity Pathway Analysis, IPA) [33]. The transcriptome microarray data are fully available from the GEO (Gene Expression Omnibus) database [34] under accession numbers GSE49694 (*Cox4i2* ^*tm1Hutt*^), GSE39953 (*Ifit2* ^*tm1*.*1Ebsb*^), and GSE49705 (*Prdm11* ^*tm1*.*1ahl*^).

## Results

### Selection of mouse models for OVA airway challenge

To select new mutant mouse lines for an altered airway inflammatory response we considered four selection criteria covering published information and phenotypical data from the primary screen of the German Mouse Clinic (GMC) (Table 1). First, we searched public databases and the relevant literature for associations of the mutated genes of previously GMC phenotyped mutant mouse lines with inflammation. Second, we assessed whether the mutated genes are expressed in lungs of wild type mice either based on original data from the primary GMC screen or based on information from public databases or literature. Third, we specifically selected mutant mouse lines without a detectable immunological phenotype in the primary GMC phenotyping screen under standard, non-challenging husbandry conditions. Finally, we took into account whether transcriptome data from lung obtained in the primary GMC screen revealed regulated genes that were associated with an inflammatory response.

**Table 1:** Criteria for selection of mutant mouse line.

Selection criterion	*Cox4i2*	*Ifit2*	*Prdm11*
**Association with inflammatory response in published data**	No, but pulmonary function [19]	Yes [20]	No, but pulmonary function [40]
**Gene expression in lung**	Yes^a^	Yes^b^	Yes^a^
**^b^Absence of immunological changes under non-challenging conditions**	Yes	Yes	Yes
**^b^Transcriptome analysis of lung pointing to inflammation**	Yes	No	Yes

^a^published data

^b^original data from GMC screen

Regarding the first criterion, *Cox4i2*, an isoform of the terminal enzyme of the respiratory chain expressed in lung, is required for normal pulmonary function. Since its loss of function led to impaired airway constriction and reduced airway responsiveness, it was suggested as a potential new target for the treatment of asthma [19]. *Ifit2*, a gene involved in cellular apoptosis [35] and viral defense [36–38], functions in the regulation of the proinflammatory cytokine response [20]. *Prdm11*, a tumour suppressor gene in MYC-driven B cell lymphomas [21] and involved in transcriptional regulation [39], was recently associated with pulmonary function in humans in a genome-wide association study (GWAS) [40].

The second criterion, expression in lung, was previously shown for *Cox4i2* and *Prdm11*. *Cox4i2* is strongly expressed in smooth muscle and lining epithelium of lung [41]. *Prdm11* was recently identified as a gene involved in lung development [40] and high levels of expression in lung were shown previously [21]. To our knowledge, expression of *Ifit2* in lung has not been reported yet. However, our own transcriptome analysis establishes *Ifit2* expression in lung (S1 Fig).

**Fig 1:**
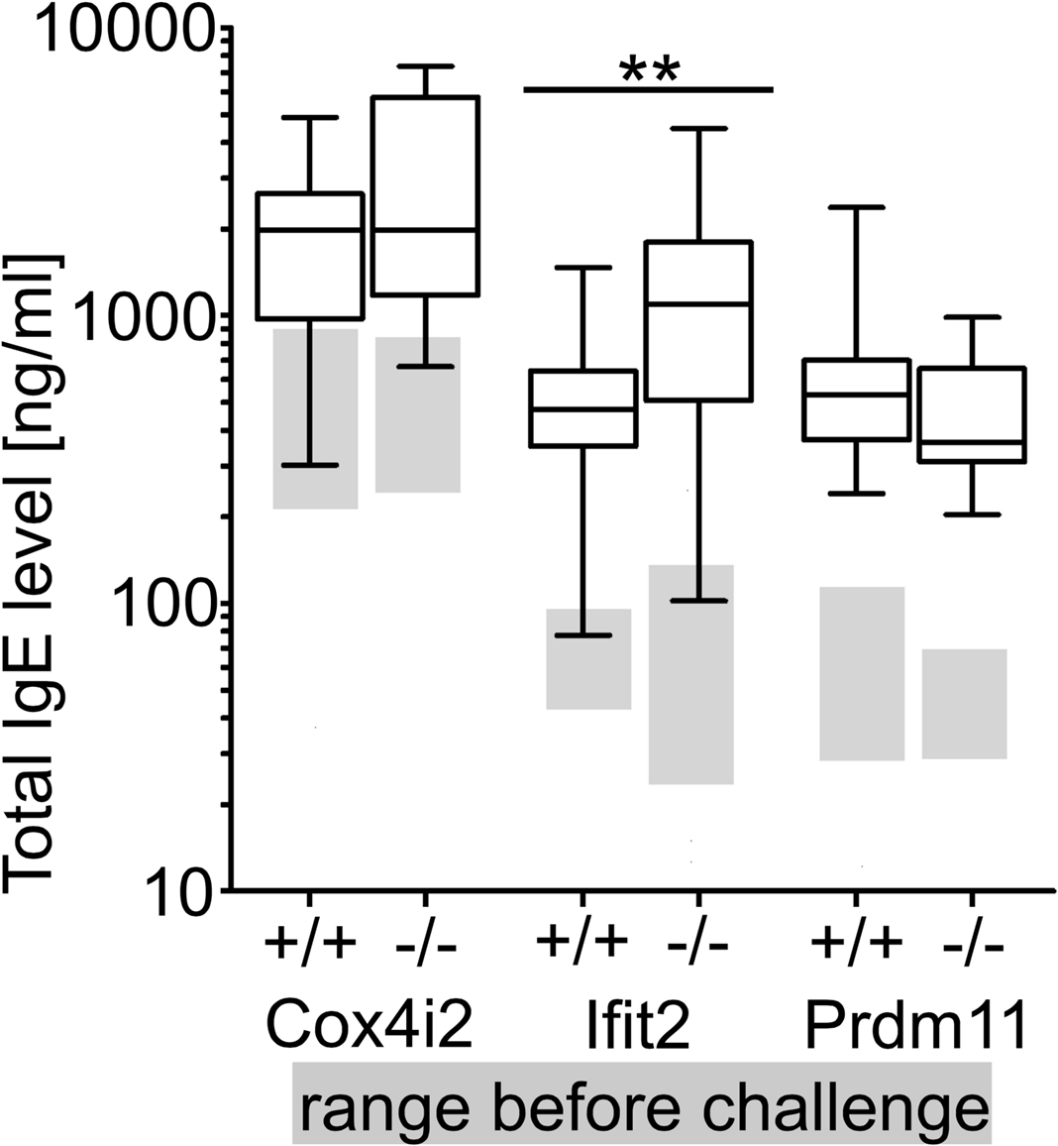
Total IgE levels in plasma before and after OVA challenge. Box plots show total IgE levels after OVA challenge in mutant mouse lines and corresponding wild type littermates. Grey boxes show the interquartile ranges of total IgE levels under standard conditions of the respective groups (p-value ** < 0.01, n = 11–31 per group).

Our third criterion, the absence of an immunological phenotype under non-challenging husbandry conditions, was also fulfilled by all three selected mutant mouse lines. In the primary GMC screen, total IgE and additional immunoglobulin levels were measured in blood and did not differ between *Cox4i2* ^*tm1Hutt*^, *Ifit2* ^*tm1*.*1Ebsb*^, and *Prdm11* ^*tm1*.*1ahl*^ mutant and the respective wild type littermates (grey bars in Fig 1 and data not shown). Thus, the primary GMC screen under standard husbandry conditions did not provide evidence for a role of the selected genes in inflammation.

### Transcriptome analyses of unchallenged mutant mice

**Fig 2:**
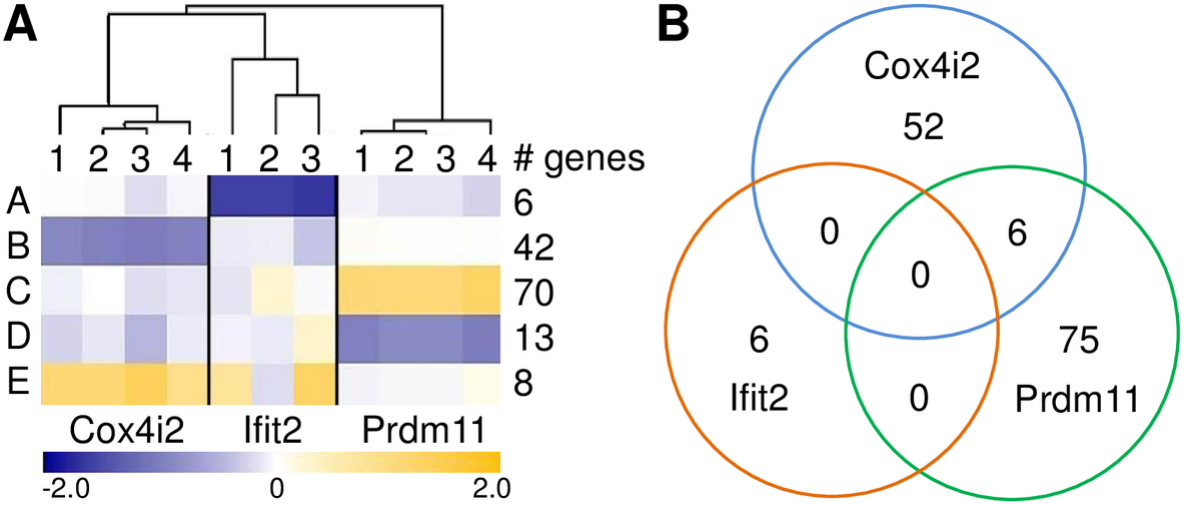
Comparative analysis of transcriptome data under standard conditions. (A) Summarized heat map from HCL analysis of genes regulated in the three mutant mouse lines. Genes with similar expression patterns are grouped together (A to E). Color code indicates the mean fold change of the respective genes in one group for each mutant mouse compared to the mean of the corresponding wild type littermate group. Orange represents up- and blue down-regulation in the respective mutant mice. The column “# genes” gives the number of genes that are included in each group A to E. (B) Venn diagram of differentially expressed genes common or specific in *Cox4i2* ^*tm1Hutt*^, *Ifit2* ^*tm1*.*1Ebsb*^ and *Prdm11* ^*tm1*.*1ahl*^ mutant mice.

Finally, by performing differential transcriptome analyses of lungs from mutant and wild type control littermates, we addressed the fourth criterion. Under non-challenging conditions, Significant Analysis of Microarrays (SAM) detected 58 differentially expressed genes in *Cox4i2* ^*tm1Hutt*^, 6 in *Ifit2* ^*tm1*.*1Ebsb*^ and 81 in *Prdm11* ^*tm1*.*1ahl*^ mutant mice in comparison to the respective wild type groups with a false discovery rate (FDR) below 10% and a mean fold change of more than 1.7 fold (S1 Fig). Hierarchical cluster analysis (HCL) of the regulated genes grouped respective mutant mice closely together and displayed genotype-specific expression patterns (Fig 2A). Nevertheless, six genes were regulated in both the *Cox4i2* ^*tm1Hutt*^ and the *Prdm11* ^*tm1*.*1ahl*^ mutant mouse lines (Fig 2B). Whereas the genes *Plcb2*, *Tmem87a*, and *9130008F23Rik* were regulated in the same direction in both mouse lines, *Csrnp1*, *Dkk1*, and *Golga2* were down-regulated in *Cox4i2* ^*tm1Hutt*^ mice and up-regulated in *Prdm11* ^*tm1*.*1ahl*^ mice (S1 Fig). *Plcb2* has been shown to influence the regulation of macrophage functions [42], whereas no function in the inflammatory response has been described for any of the other five genes.

Due to the low number of regulated genes in lungs of *Ifit2* ^*tm1*.*1Ebsb*^ mice a bioinformatics Gene Ontology (GO) term enrichment analysis was not appropriate for this mutant line. However, we noted, in particular, the reduced expression of *Hermansky-Pudlak syndrome 1 homolog* (*Hps1*). Symptoms of Hermansky-Pudlak syndrome in humans include pulmonary fibrosis [43]. The differentially expressed genes in lungs of the other two mutant mouse lines were classified according to over-represented GO annotations. In *Cox4i2* ^*tm1Hutt*^ over-represented terms were almost exclusively associated with an inflammatory response and the differentiation and migration of immune cells (Table 2A). In *Prdm11* ^*tm1*.*1ahl*^ mutant mice over-represented GO terms also belonged to the category inflammatory response but, in addition, included genes with functional annotations of the categories carbohydrate and nucleic acid metabolism, and tissue development (Table 2B). Thus, the functional classifications of differentially expressed genes in lungs of *Cox4i2* ^*tm1Hutt*^ and *Prdm11* ^*tm1*.*1ahl*^ mutant mouse lines in comparison to wild type littermates point towards a potential predisposition for an altered inflammatory response in the respective mutant mice.

**Table 2:** Over-represented GO functional annotations of regulated genes in A) *Cox4i2* ^tm1Hutt^ and B) *Prdm11* ^*tm1*.*1ahl*^ mutant mice under standard husbandry.

**A Category**	**Functional annotation**	**p-value**	**# genes**
Cellular development	development of lymphocytes	8.17E-03	6
Cellular movement	cell movement of neutrophils	2.14E-04	6
	chemotaxis	5.45E-03	6
	migration of cells	1.30E-02	12
Cell-to-cell signaling	activation of cells	5.17E-03	8
Hematological system	quantity of neutrophils	6.78E-05	6
	differentiation of hematopoietic progenitor cells	8.45E-05	6
	differentiation of blood cells	1.55E-02	7
	quantity of leukocytes	1.91E-03	10
Immunological disease	hypersensitive reaction	9.54E-04	7
	systemic autoimmune syndrome	7.88E-04	11
Inflammatory response	inflammation of lung	1.70E-03	6
	inflammatory response	4.54E-03	7
Organismal injury	fibrosis	6.89E-03	6
**B Category**	**Functional annotation**	**p-value**	**# genes**
Carbohydrate metabolism	uptake of monosaccharide	4.14E-02	4
Cellular growth and proliferation	formation of cells	1.88E-02	6
Free radical scavenging	synthesis of reactive oxygen species	5.17E-03	8
Hematological system	homeostasis of blood	2.93E-03	3
Inflammatory response	inflammatory response	2.74E-02	8
	inflammation of body region	2.95E-02	9
Muscular disorders	congenital anomaly of musculoskeletal system	6.50E-04	10
Nucleic acid metabolism	metabolism of nucleic acid component	1.06E-02	8
Tissue development	growth of epithelial tissue	3.59E-02	7

### Immunological effects in OVA challenged mutant mice

**Fig 3:**
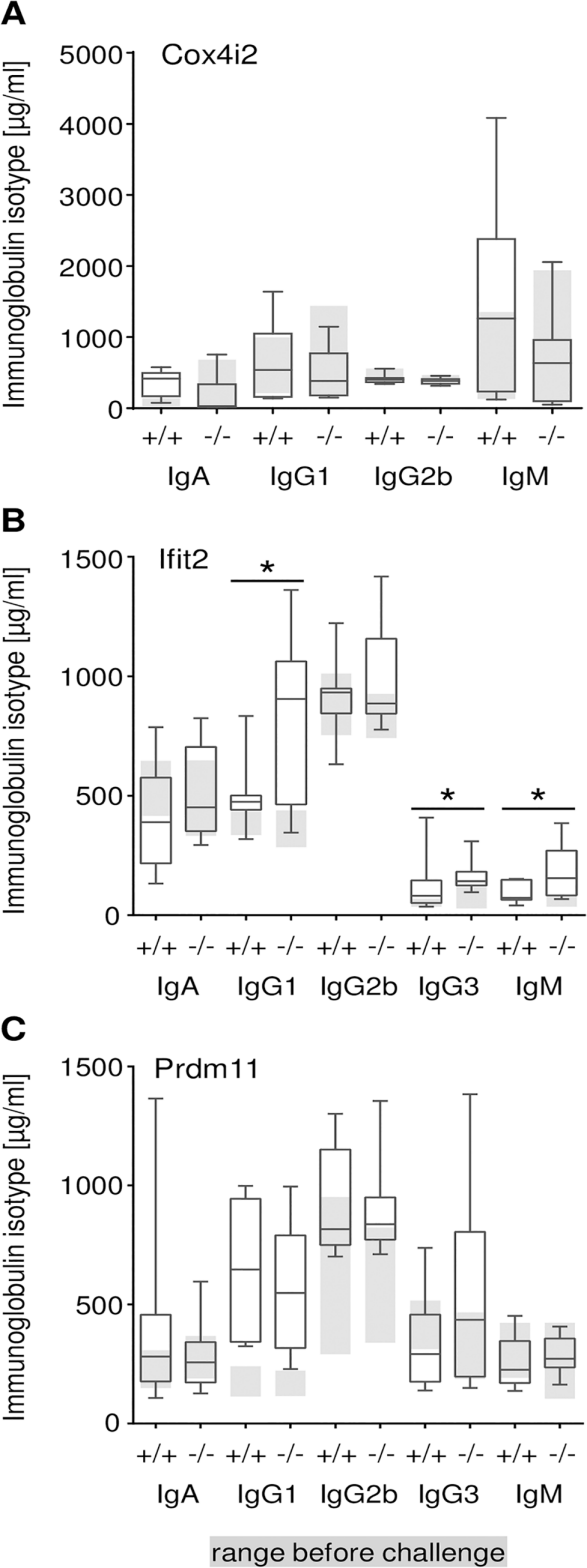
Immunoglobulin isotype levels in mutant mice before and after OVA challenge. **(**A) *Cox4i2* ^*tm1Hutt*^, (B) *Ifit2* ^*tm1*.*1Ebsb*^ and (C) *Prdm11* ^*tm1*.*1ahl*^. Box plots show levels of immunoglobulin isotypes in the respective mutant mice and corresponding wild type littermates after OVA challenge. Grey boxes display the interquartile range of each immunoglobulin isotype under standard conditions (+/+: wild type,-/-: respective mutant mice; p-value * < 0.05; n = 10–12 per group).

**Fig 4:**
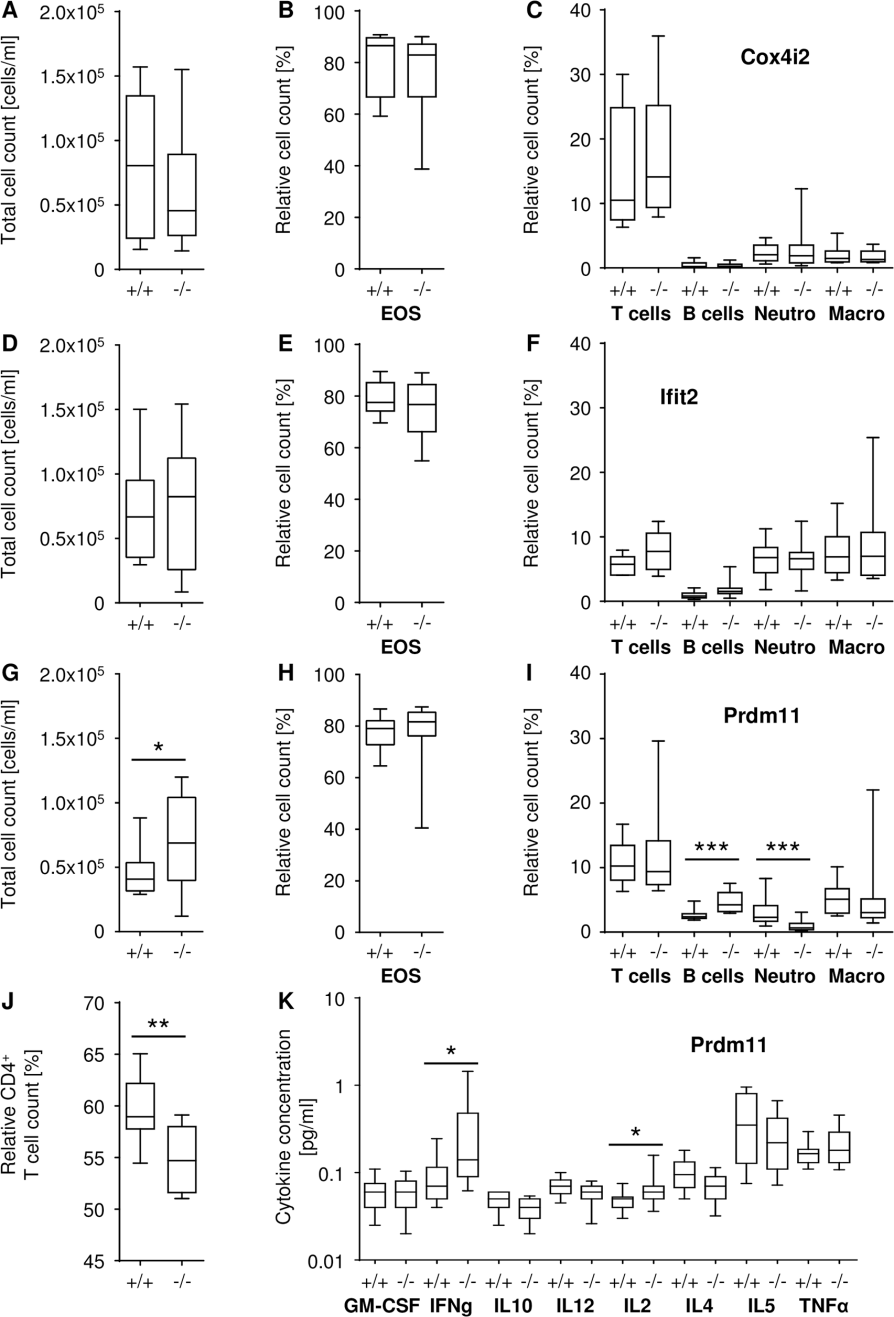
Cytometric and cytokine analyses in BAL after OVA challenge. (A to C) *Cox4i2* ^*tm1Hutt*^, (D to F) *Ifit2* ^*tm1*.*1Ebsb*^ and (G to K) *Prdm11* ^*tm1*.*1ahl*^. (A, D, and G) Total cell count in BAL following OVA challenge. (B, C, E, F, H, and I) Relative cell count of eosinophils (Eos), T cells, B cells, neutrophils (Neutro) and macrophages (Macro) as indicated in panels following OVA challenge. (J) Relative cell count of CD4^+^ T cells in BAL following OVA challenge. (K) Quantification of cytokines in BAL following OVA challenge (p-value * < 0.05; ** < 0.01; *** < 0.001; n = 10–12 per group).

In order to evaluate the effects of the mutations in the *Cox4i2*, *Ifit2*, and *Prdm11* genes under challenge conditions, we analyzed mutant and corresponding wild type mice after OVA sensitization and subsequent OVA aerosol challenge. In the comparison of challenged *Cox4i2* ^*tm1Hutt*^ mutant versus challenged wild type littermates, no statistically significant alterations were detected in the levels of total IgE (Fig 1), immunoglobulin isotypes (Fig 3A), and cell populations in BAL (Fig 4A–4C) [19].

**Fig 5:**
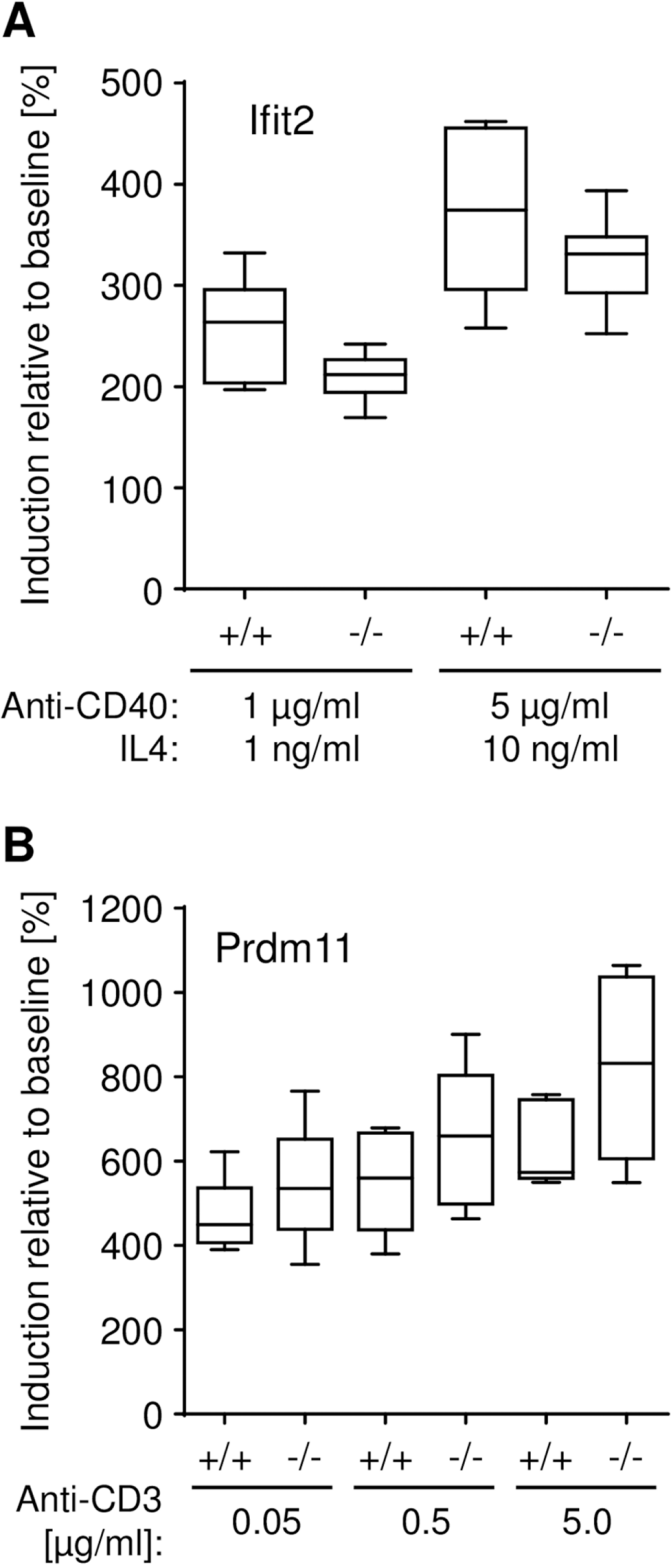
*In vitro* splenocyte proliferation. *In vitro* proliferation response was investigated in *Ifit2* ^*tm1*.*1Ebsb*^ and *Prdm11* ^*tm1*.*1ahl*^ splenocyte cultures. (A) For B-cell activation, *Ifit2* ^*tm1*.*1Ebsb*^ splenocytes were cultured with 1 μg/ml anti-CD40 and 1 ng/ml IL-4 or 5 μg/ml anti-CD40 and 10 ng/ml IL-4 (n = 6 per group). (B) For T-cell stimulation, *Prdm11* ^*tm1*.*1ahl*^ splenocytes were cultured with 20 U/ml IL-2 and either 0.05 μg/ml, 0.5 μg/ml or 5.0 μg/ml of anti-CD3 for T cell activation (n = 5 per group).

In contrast, statistically significant alterations were detected in *Ifit2* ^*tm1*.*1Ebsb*^ mutant mice. These included increased levels of IgE (Fig 1), IgG1, IgG3, and IgM (Fig 3B) in mutant compared to control mice under challenge conditions. Moreover, a slight increase of CD3^+^ cells (p<0.02) and the Ly6c^+^ fraction from the CD4^+^ T cells (p<0.03) was detected in challenged *Ifit2* ^*tm1*.*1Ebsb*^ mutant mice in comparison to the wild type littermates (data not shown). Due to the increased immunoglobulin levels in *Ifit2* ^*tm1*.*1Ebsb*^ mutant mice, we tested the activation and immunoglobulin class switching of B cells as well as the proliferation of T cells by *in vitro* stimulation of splenocytes from an independent and unchallenged cohort of mice. Neither B nor T cells showed differences in activation or proliferation under cell culture conditions (Fig 5A). Cell populations in BAL following OVA challenge were not different in *Ifit2* ^*tm1*.*1Ebsb*^ mutant and respective control mice (Fig 4D–4F).

Challenged *Prdm11* ^*tm1*.*1ahl*^ mutant mice did not show differences in IgE levels (Fig 1) or other immunoglobulin levels in comparison to challenged wild type littermates (Fig 3C). However, a significant increase in the total cell number collected from BAL was found in these mutant mice (Fig 4G). Proportions of eosinophils (Fig 4H), T cells and macrophages were unchanged (Fig 4I). In contrast, proportions of neutrophils were highly significantly decreased, whereas relative B cell counts were highly significantly increased (Fig 4I) in *Prdm11* ^*tm1*.*1ahl*^ mutant mice. The CD4^+^ T cell subpopulation in BAL from *Prdm11* ^*tm1*.*1ahl*^ mutant mice was significantly decreased compared to the challenged control animals (Fig 4J). In BAL from *Prdm11* ^*tm1*.*1ahl*^ mutant mice after challenge we measured slightly increased concentrations of IFNg and IL2, whereas other cytokines (GM-CSF, IL10, IL12, IL4, IL5, and TNFa) were unchanged in the comparison betwenn mutant and wild type mice (Fig 4K). Moreover, the *in vitro* proliferation assays for stimulation of T cells revealed consistent trends towards increased total splenocyte counts in *Prdm11* ^*tm1*.*1ahl*^ mutants after stimulation with Il-2 and different concentrations of anti-CD3 (Fig 5B).

Taken together, these results describe distinct immune phenotypes for *Prdm11* ^*tm1*.*1ahl*^ and *Ifit2* ^*tm1*.*1Ebsb*^ mutant mice under OVA challenge conditions and suggest functional roles for both genes in the airway inflammatory response.

### Transcriptomes in OVA challenged mutant mice

To evaluate changes in gene expression patterns under OVA airway challenge conditions, we performed differential transcriptome analyses of lungs from challenged mutant mice and the corresponding challenged littermate control mice. In total, 169 differentially expressed genes with a fold change > 1.7 fold and an FDR < 10% were detected in *Cox4i2* ^*tm1Hutt*^ (S2A Fig), 175 regulated genes in *Ifit2* ^*tm1*.*1Ebsb*^ (S2B Fig), and 3078 regulated genes in *Prdm11* ^*tm1*.*1ahl*^ (S2C Fig). Although changes in transcriptomes were extensive in all three challenged mutant lines, the difference in the mere number of regulated genes in the *Prdm11* mutant mice as compared to those in the *Cox4i2* and *Ifit2* mutant mice is striking.

With one exception (*Lmo3*), genes in lungs of the *Cox4i2* ^*tm1Hutt*^ mutant mouse line were all down-regulated. Among the top 20 genes with a mean reduced expression of 10 to 67 fold in the comparison to challenged wild type littermates are almost exclusively muscle specific genes (*Acta1*, *Tnnc1*, *Myh2*, *Myh8*, *Tpm2*, *Mylpf*, *Myl2*, *Myl1*, *Mb*, and several others related to keratin, see S2A Fig). Similarly, the top down-regulated genes in the *Ifit2* ^*tm1*.*1Ebsb*^ mutant mouse line also include a significant number of muscle and keratin associated genes (*Myl2*, *Krt13*, *Myh2*, *Krt4*, *Myoz1*, *Mb* and others, see S2B Fig), although mean fold changes of the top 20 down-regulated genes in this mutant mouse line are more modest, ranging from 4 to 15 fold.

One remarkable finding in the *Prdm11* ^*tm1*.*1ahl*^ mutant mouse line is the more than 300 fold up-regulation of *TNFAIP3 interacting protein 2* (*Tnip2*, synonym *Abin-2*) in comparison to the challenged wild type littermates (S2C Fig). Interestingly, it was previously shown that *Tnip2* is required for an optimal activation of the innate immune response upon LPS induction [44]. All other up-regulated genes in this mutant mouse line had mean fold changes less than 8 fold.

**Fig 6:**
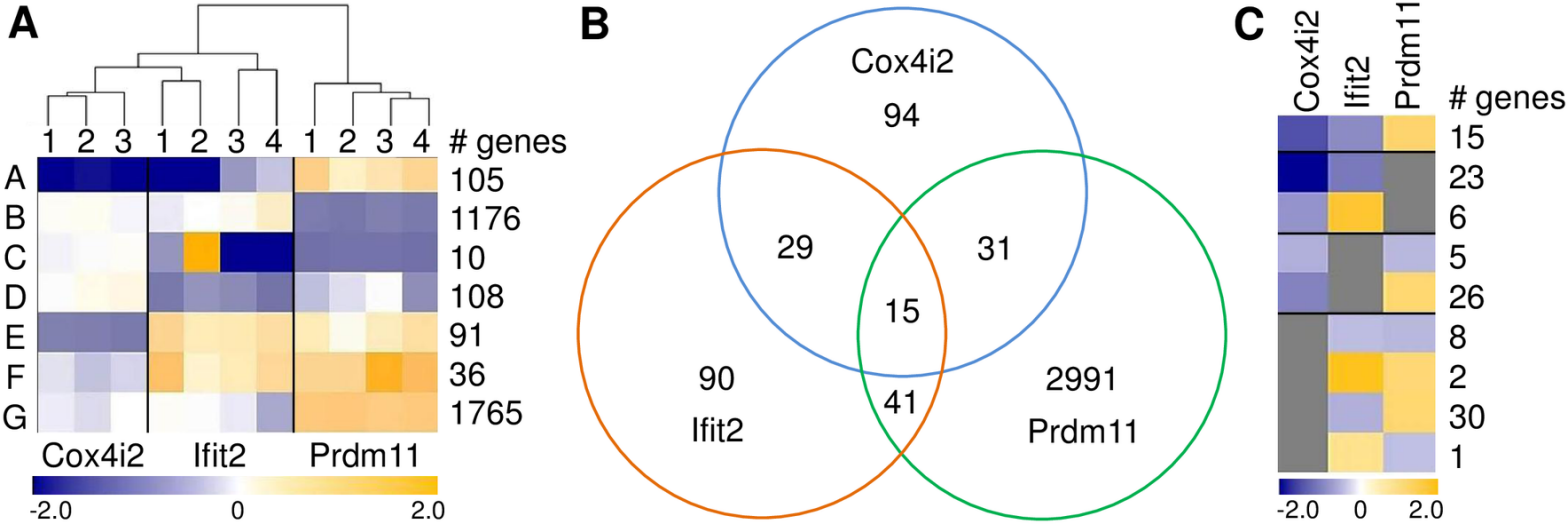
Lung transcriptome comparison of mutant versus wild type animals following OVA challenge. (A) Summarized heat map of genes regulated after OVA challenge in all three mutant mouse lines compared to the respective challenged control littermates. Genes with similar expression patterns are grouped together (A to G). Color code indicates the mean fold changes of the respective genes in one group for each OVA challenged mutant mouse compared to the mean of the corresponding challenged wild type littermates. Orange represents up- and blue down-regulation in the mutant mice. The column “# genes” displays the number of genes that are included in each gene group A to G (S2D Fig shows the corresponding data for individual genes). (B) Venn diagram representing the overlap of gene regulation. (C) Summarized heat map of overlapping gene expression in at least 2 of the 3 mutant mouse lines. Grey boxes indicate that there is no significant regulation of these genes in the respective mutant mouse lines (S2E Fig shows the data for individual genes of this panel).

For the comprehensive analysis of transcriptional changes between the challenged mutant mouse lines and challenged wild type littermates, we searched for over-represented GO terms (Table 3) and performed an HCL analysis (Fig 6A). Functional annotations associated with an altered immune or inflammatory response were over-represented in the differential transcriptome analyses of all three challenged mutant mouse lines, suggesting that the knock-outs of *Cox4i2*, *Ifit2*, and *Prdm11* each affect the inflammatory response. However, the most significant association of regulated genes with immune cell proliferation was evident in lungs of the challenged *Prdm11* ^*tm1*.*1ahl*^ mutant mouse line, not only in terms of the number of regulated genes but also with regards to p-values (see, for example, 127 regulated genes associated with “proliferation of T lymphocytes” with p < 8.76 x 10^−6^, Table 3C). In addition, we noted the over-representation of differentially expressed genes associated with carbohydrate and/or lipid metabolism in all three mutant mouse lines (Table 3).

**Table 3:** Over-represented GO functional annotations of regulated genes in A) *Cox4i2* ^*tm1Hutt*^, B) *Ifit2* ^*tm1*.*1Ebsb*^ and C) *Prdm11* ^*tm1*.*1ahl*^ mutant mice under allergen challenge conditions.

**A Category**	**Functional annotation**	**p-value**	**# genes**
Carbohydrate metabolism	metabolism of carbohydrate	6.83E-03	11
Cell morphology	size of cells	4.20E-03	9
Cellular organization	formation of filaments	2.57E-02	7
Cellular movement	lymphocyte migration	1.46E-02	7
	migration of phagocytes	1.31E-02	6
Immunological disease	systemic autoimmune syndrome	1.85E-02	17
	hypersensitive reaction	1.08E-02	10
	allergy	1.87E-02	9
	dermatitis	8.69E-03	10
Lipid metabolism	concentration of lipid	1.72E-02	14
**B Category**	**Functional annotation**	**p-value**	**# genes**
Cellular movement	migration of cells	3.50E-02	22
Immunological disease	atopic dermatitis	3.28E-02	6
	immediate hypersensitivity	2.69E-02	7
	allergy	3.02E-02	8
Lipid metabolism Organismal injury	concentration of lipid	1.92E-02	13
	fibrosis	1.07E-03	12
Tissue development	development of muscle	2.47E-03	9
**C Category**	**Functional annotation**	**p-value**	**# genes**
Carbohydrate metabolism	uptake of carbohydrate	3.46E-03	44
Cell death and survival	apoptosis of lymphocytes	2.16E-03	70
	cell death of epithelial cells	8.80E-03	77
	apoptosis of leukocytes	1.37E-03	90
Cellular movement	NK cell migration	2.50E-03	11
	cell movement of epithelial cells	2.83E-03	25
	cell movement of granulocytes	5.77E-03	69
	cell movement of lymphocytes	7.96E-03	69
	cell movement of phagocytes	8.16E-03	95
	cell movement of leukocytes	8.17E-03	134
Cellular proliferation	proliferation of fibroblasts	7.43E-04	62
	proliferation of T lymphocytes	8.76E-06	127
	proliferation of lymphocytes	1.21E-05	151
Gene expression	expression of RNA	2.40E-06	407
Hematological system	development of B lymphocytes	4.67E-03	26
	differentiation of lymphocytes	4.94E-03	92
	quantity of leukocytes	1.93E-03	188
Immune response	quantity of IgM	6.51E-03	32
Inflammatory response	degranulation of mast cells	2.79E-04	28
	degranulation of phagocytes	6.80E-05	32
	accumulation of leukocytes	6.58E-03	45
Lipid metabolism	concentration of lipid	4.71E-04	159

HCL of the differentially regulated genes between mutant lines and wild type littermates under challenge conditions classified seven groups of genes based on similar expression patterns (gene groups A to G in Fig 6A) and clearly separated *Prdm11* ^*tm1*.*1ahl*^ mice from the other two mutant mouse lines (Fig 6A and S2D Fig). In particular, 44 genes were regulated in both the *Cox4i2* ^*tm1Hutt*^ and the *Ifit2* ^*tm1*.*1Ebsb*^ mutant mouse lines (Fig 6B and top three rows in Fig 6C). Out of these 44 genes, the majority (38 genes) showed similar patterns of down-regulation in both mutant mouse lines (top two rows in Fig 6C, and S2E Fig). Fifteen genes that were regulated in all three challenged mutant mouse lines (Fig 6B), were down-regulated in lungs of challenged *Cox4i2* ^*tm1Hutt*^ and *Ifit2* ^*tm1*.*1Ebsb*^ mutant mice and were up-regulated in challenged *Prdm11* ^*tm1*.*1ahl*^ mutant mice (top row in Fig 6C).

Overall, the differential transcriptome analysis of challenged mutant and challenged wild type mice provides evidence for a role of all three genes examined, *Cox4i2*, *Ifit2*, and *Prdm11*, in the airway inflammatory response.

## Discussion

Co-ordinated by the International Mouse Phenotyping Consortium (IMPC), mouse phenotyping centers around the world currently aim at the systematic broad-based phenotyping of each protein-coding gene in the mouse genome [45, 46]. For the first time for a mammalian genome, the scientific community will soon have access to primary phenotyping data and gene functional annotations for every protein-coding gene in the mouse. To reach this goal the contributing phenotyping centers have developed standardized husbandry conditions and phenotyping screens [47]. However, in this work here we considered the notion that mutant mouse lines might not manifest an altered phenotype under standard husbandry conditions, due to the absence of a relevant environmental challenge [16]. Based on original primary phenotyping data from the German Mouse Clinic and published data, we aimed at the effective selection of potential mouse models for an altered airway inflammatory response following OVA sensitization and challenge from more than 300 phenotyped mutant mouse lines. As conditio sine qua non we considered only mutant mouse lines of genes that are expressed in lung, the primary target organ of the OVA challenge (Table 1). To uncover mutant phenotypes that only become apparent under challenge conditions, we exclusively selected mutant lines that were inconspicuous in the primary immunology and inflammation screens under standard conditions. In addition, we required that the selected mutant mouse lines were previously associated with an altered inflammatory response in published data (*Ifit2* ^*tm1*.*1Ebsb*^) or had gene expression changes under non-challenging conditions (*Cox4i2* ^*tm1Hutt*^ and *Prdm11* ^*tm1*.*1ahl*^, see Fig 2 and Table 2). The subsequent phenotypic analysis revealed an altered cellular immune response for the challenged *Prdm11* ^*tm1*.*1ahl*^ mutant mice and *Ifit2* ^*tm1*.*1Ebsb*^ mutant mice were affected in their humoral immune response. In contrast, *Cox4i2* ^*tm1Hutt*^ mutant mice neither revealed an altered cellular nor humoral immune response in our assays. However, all three selected mutant lines showed clear genotype specific differences in their lung transcriptomes after OVA challenge. In this regard we consider our selection strategy as highly effective in identifying genes required for a normal airway inflammatory response. In addition, our data highlight the importance of primary phenotyping data as a basis for the selection of specific mouse models for subsequent focused gene functional studies, and show the usefulness of environmental challenge studies for revealing genetic predispositions and altered phenotypes that do not manifest under standard husbandry consitions.

OVA sensitization followed by repeated OVA challenges in rodent models as well as chronic asthma in humans induce extensive airway remodeling including thickening of the airway smooth muscle (ASM) layer [48–50]. Our gene expression analysis suggests that ASM remodeling is one of the major processes that is altered in all three mutant mouse lines examined. However, whereas a group of muscle associated genes is strongly reduced in expression levels in both the *Ifit2* ^*tm1*.*1Ebsb*^ and *Cox4i2* ^*tm1Hutt*^ mutant mice, these genes are either increased in their expression levels in *Prdm11* ^*tm1*.*1ahl*^ mutant mice (*Myoz1*, *Tmod4*, *Tpm2*) or not differentially expressed there (*Mb*, *Myh2*, *Myl2*) (see Figs 6C and S2E). Interestingly, we observed that a group of genes with known functions in the cornified envelope and previously associated with ichthyosis [51] follow the same expression pattern in the individual mutant mouse lines as the muscle associated genes. This includes, in particular, several members of the *late cornified envelope* and *keratin* gene families, as well as *Rptn*, *Hrnr*, and *Cnfn* (see Figs 6C and S2E). These data, together with our results of the HCL analysis (Fig 6A), show a major differences in the altered airway inflammatory response in the *Prdm11* mutant mice on the one hand and the *Cox4i2* and *Ifit2* mutant mice on the other hand.

Differences between all three mutant lines following the OVA challenge were also evident in the immunological parameters that we measured. Only *Ifit2* ^*tm1*.*1Ebsb*^ mutant mice showed clear alterations in the humoral immune response (Fig 1). In particular, IgE, IgG1, IgG3 and IgM levels in plasma of challenged *Ifit2* ^*tm1*.*1Ebsb*^ mutant mice were elevated in comparison to the challenged wild type littermates (Figs 1 and 3B). In contrast, *Prdm11* ^*tm1*.*1ahl*^ mutant mice had an affected cellular immune response. Following the OVA challenge, total cell counts in BAL were elevated in *Prdm11* ^*tm1*.*1ahl*^ mutant mice compared to the challenged wild type littermates (Fig 4G) and the relative counts of neutrophils, B cells (Fig 4I) and CD4^+^ T cells in BAL (Fig 4J) were altered in this comparison. Despite the conspicuous changes in transcriptomes between challenged *Cox4i2* ^*tm1Hutt*^ mutant mice and wild type littermates described above, we neither detected changes in the humoral nor in the cellular immune response for this mutant line. One possibility could be that this finding may be due to the fact that the *Cox4i2* mutant and wild type cohorts were submitted to the OVA airway challenge procedure at the age of eight weeks and might potentially develop an altered innate or humoral immune response later in life.

In summary, we present here an effective way of identifying new models for an altered airway inflammatory response under OVA challenge conditions from a compendium of several hundred mutant mouse lines that were inconspicuous with regards to immunological phenotypes in primary phenotyping screens. Considering the international efforts to fully annotate the first mammalian genome, analogous selection strategies may be required for the effective analysis of further envirotype–genotype interactions for other diseases.

## Supporting Information

S1 ARRIVE Checklist ARRIVE Checklist. (DOCX)

S1 Fig Transcriptional changes in lung under standard conditions. Heatmaps display significantly regulated genes *in Cox4i2* ^*tm1Hutt*^, *Ifit2* ^*tm1*.*1Ebsb*^ and *Prdm11* ^*tm1*.*1ahl*^ mice. The color code indicates the mean fold change of every regulated gene for each mutant mouse compared to the mean of the respective wild type littermate group. Orange represents up-regulation and blue down-regulation in mutant mice.
(XLSX)

S2 Fig Transcriptional changes in lung after OVA challenge. Heatmaps display significantly regulated genes in OVA challenged A) *Cox4i2* ^*tm1Hutt*^, B) *Ifit2* ^*tm1*.*1Ebsb*^, and C) *Prdm11* ^*tm1*.*1ahl*^ mice compared to the respective challenged wild type littermates. The color code indicates the fold change of every gene for each mutant sample compared to the mean of the respective control group under challenge conditions. D) HCL analysis of all regulated genes. The column designated as ‘group’ assigns a name for each group of genes with similar expression patterns as summarized in Fig 5A. Orange represents up-regulation and blue down-regulation in mutant mice. E) Heatmap of overlapping gene expression in at least 2 of the 3 mutant mouse lines as summarized in Fig 5C. Grey boxes indicate that there is no significant regulation of these genes in the respective mutant mouse lines.
(XLS)

S1 Table Description of cohorts. (DOC)
