# Supplementary material for: Cox4i2, Ifit2, and Prdm11 Mutant Mice: Effective Selection of Genes Predisposing to an Altered Airway Inflammatory Response from a Large Compendium of Mutant Mouse Lines
Source: PLoS One. 2015 Aug 11;10(8):e0134503. doi: 10.1371/journal.pone.0134503 (PMC4532500; doi:10.1371/journal.pone.0134503)

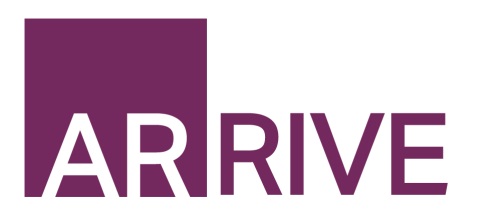


The ARRIVE Guidelines Checklist

Animal Research: Reporting In Vivo Experiments

Carol Kilkenny^1^, William J Browne^2^, Innes C Cuthill^3^, Michael Emerson^4^ and Douglas G Altman^5^

*^1^The National Centre for the Replacement, Refinement and Reduction of Animals in Research, London, UK, ^2^School of Veterinary Science, University of Bristol, Bristol, UK, ^3^School of Biological Sciences, University of Bristol, Bristol, UK, ^4^National Heart and Lung Institute, Imperial College London, UK, ^5^Centre for Statistics in Medicine, University of Oxford, Oxford, UK.*

|  | | ITEM | RECOMMENDATION | Section/ Paragraph |
| --- | --- | --- | --- | --- |
| 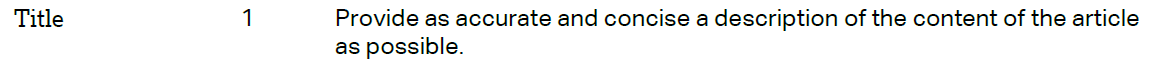 | | | page 1 , lines 1-3 |  |
| 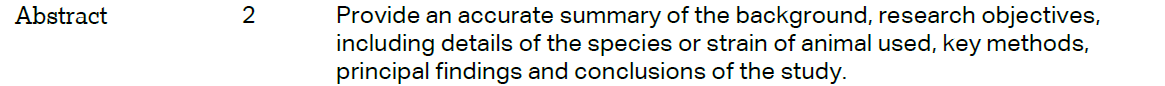 | | | page 2, lines 33-52 |  |
| INTRODUCTION | | |  |  |
| 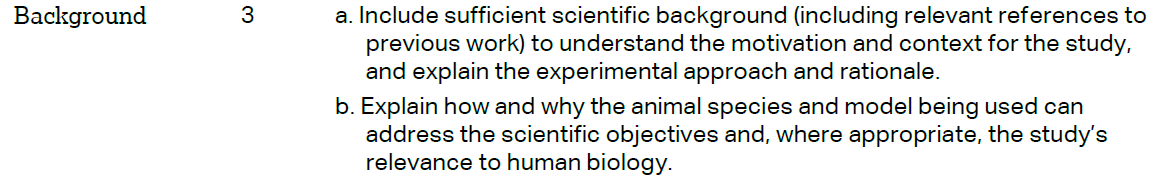 | | | a. pages 3-4, lines 54-96  b. page 4, lines 86-89 |  |
| 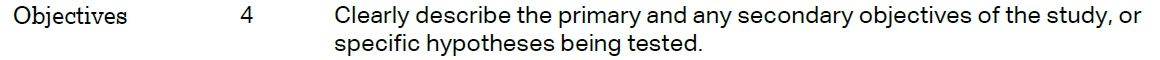 | | | page 3, lines 61-65 |  |
| METHODS | | |  |  |
| 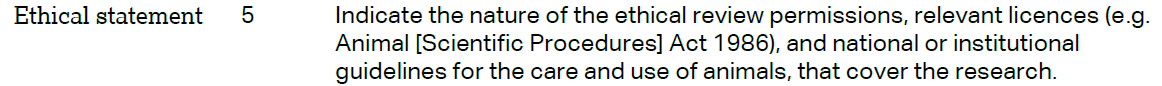 | | | page 5, lines 105-117 |  |
| 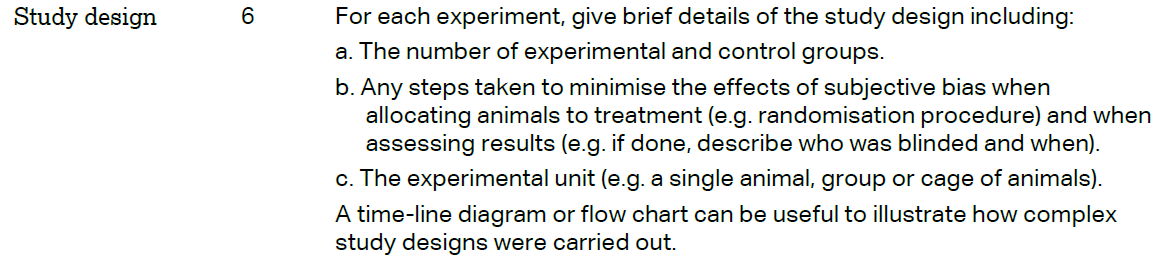 | | | S1 Table and group sizes are given in legends to figures |  |
| 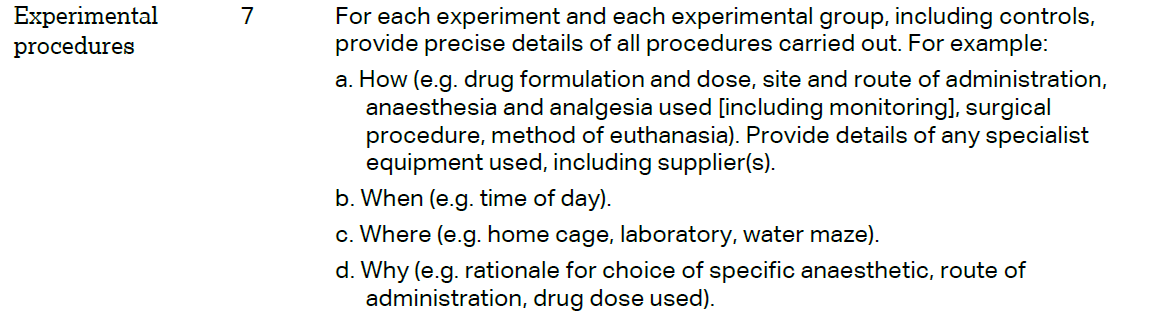 | | | page 5, lines 118-126 |  |
| 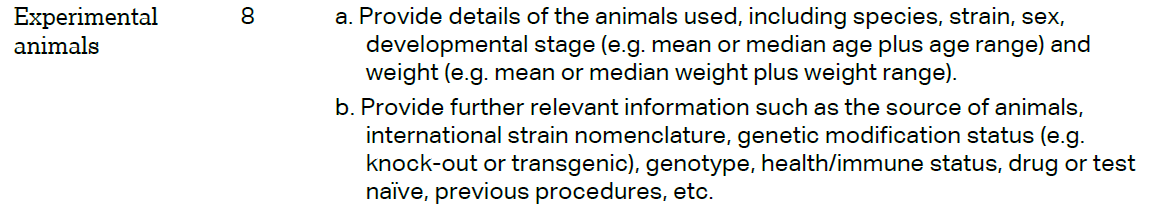 | | | page 5, lines 105-117 |  |

The ARRIVE guidelines. Originally published in *PLoS Biology*, June 2010^1^

| 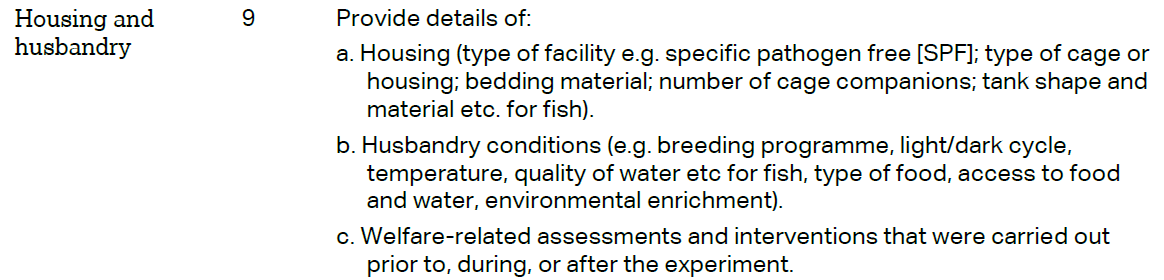 | page 5, lines 153-117 | |
| --- | --- | --- |
| 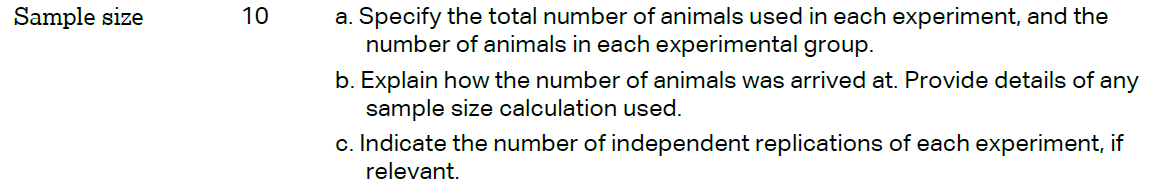 | S1 Table, page 6, lines 157-159, page 7 | |
| 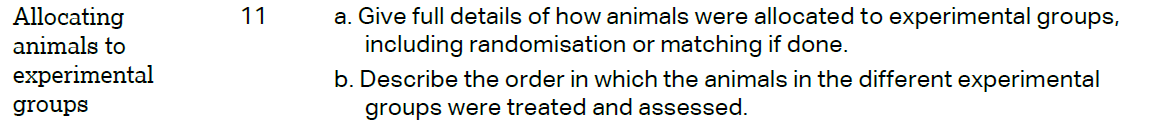 | S1 Table | |
| 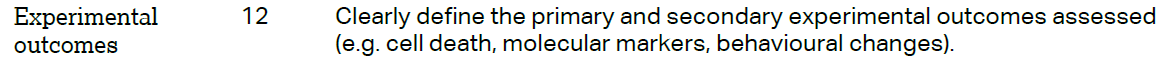 | Page 5 line 123, page 6, line 142, line 148, line 162 | |
| 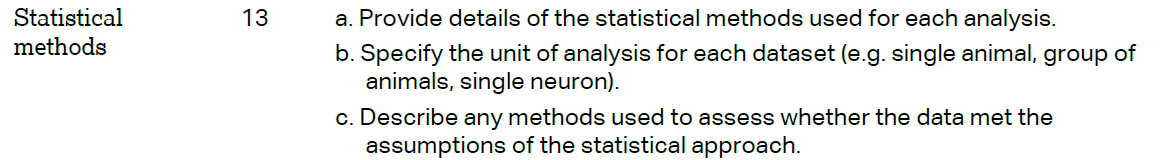 | page 7, lines 177-179 and lines 186-191 | |
| RESULTS |  | |
| 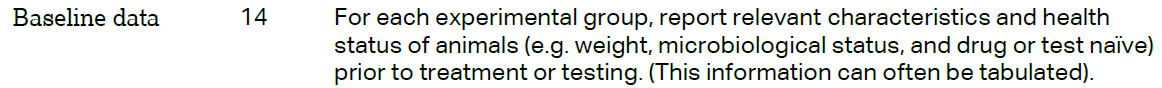 | baseline data prior treatment is represented in Figures 2 and 3 as grey bars | |
| 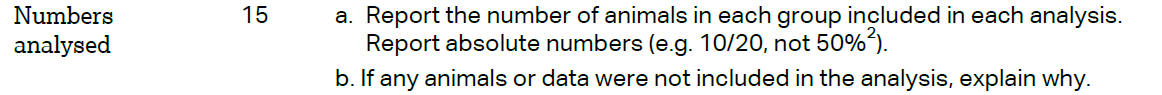 | group sizes are given in each Figure or respective legend | |
| 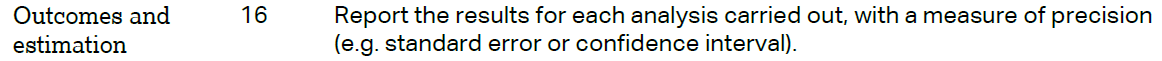 | measures of precision are given in Figures 2, 3, 4, and 5 and respective legends; for transcriptomes FDR < 10 % and FC > 1.7 | |
| 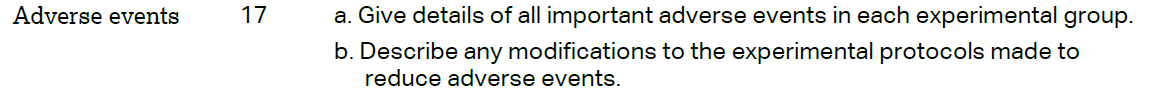 | n.a. | |
| DISCUSSION |  | |
| 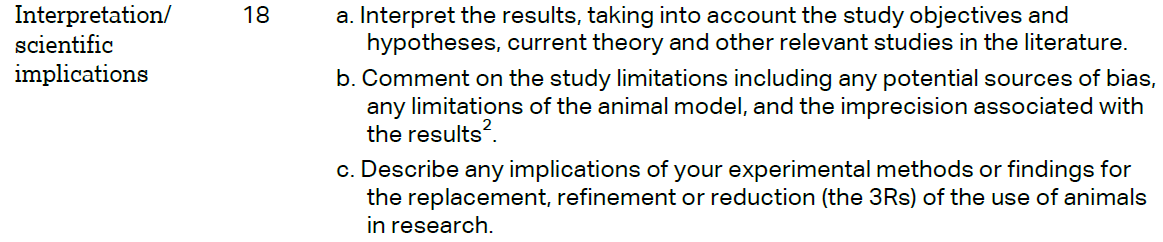 | see discussion | |
| 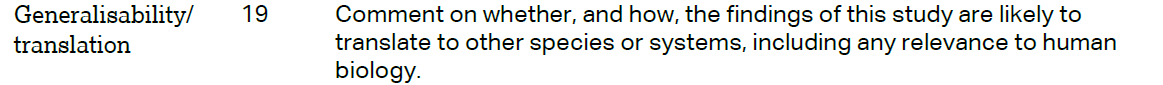 | Discussion; We provide a proof-of-concept for an effective selection strategy of mutant mice for environmental challenges that supports the first complete annotation of a mammalian genome | |
| 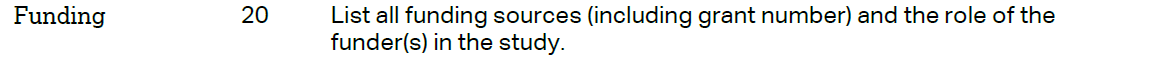 | | see financial disclosure in submission form |


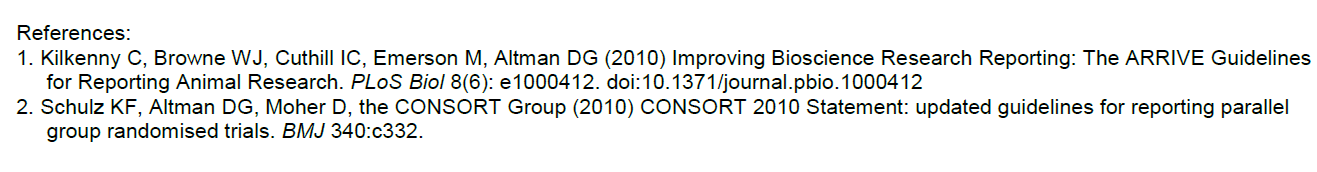

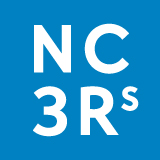

Supplement: S1 ARRIVE Checklist — (DOCX) [file pone.0134503.s001.docx]
