# Supplementary material for: Cox4i2, Ifit2, and Prdm11 Mutant Mice: Effective Selection of Genes Predisposing to an Altered Airway Inflammatory Response from a Large Compendium of Mutant Mouse Lines
Source: PLoS One. 2015 Aug 11;10(8):e0134503. doi: 10.1371/journal.pone.0134503 (PMC4532500; doi:10.1371/journal.pone.0134503)
Supplement: S1 Table — (DOC) [file pone.0134503.s004.doc]

| **cohorts** | **allele** | **genotype** | **background strain** | **age**^b^  [weeks] | **gender** | **challenged**  [group size] | **unchallenged**^c^  [group size] |
| --- | --- | --- | --- | --- | --- | --- | --- |
| **Cox4i2** | Cox4i2^tm1Hutt^ | -/- | C57BL/6 | 8 | female | 12 | 4 |
| **control**^a^ | wild type | +/+ |  | 8 | female | 12 | 4 |
| **Ifit2** | Ifit2^tm1.1Ebsb^ | -/- | C57BL/6 x  129Ola | 11 | female | 12 | 3 |
| **control**^a^ | wild type | +/+ |  | 11 | female | 11 | 4 |
| **Prdm11** | Prdm11^tm1.1ahl^ | -/- | C57BL/6 x 129Ola | 17 | female | 15 | 4 |
| **control**^a^ | wild type | +/+ |  | 17 | female | 14 | 4 |

**Table S1:** Description of cohorts

^a^wild type littermates of respective mutant line; ^b^start of OVA airway challenge; ^c^reference group for expression profiling
